# Low back pain: a major global problem for which the chiropractic profession needs to take more care

**DOI:** 10.1186/s12998-018-0199-6

**Published:** 2018-06-25

**Authors:** Simon D. French, Aron S. Downie, Bruce F. Walker

**Affiliations:** 1 Department of Chiropractic, Faculty of Science and Engineering, Macquarie University, Sydney, 2109 NSW Australia; 2 School of Rehabilitation Therapy, Queen’s University, Kingston, ON Canada; 3 University of Sydney, Sydney, Australia; 4 School of Health Professions, Murdoch University, Perth, Australia

## Abstract

An important series of papers have been published in the *Lancet*. These papers provide a comprehensive update for the major global problem of low back pain, and the challenges that low back pain presents to healthcare practitioners and policy makers. Chiropractors are well placed to reduce the burden of low back pain, but not all that chiropractors do is supported by robust, contemporary evidence. This commentary summarises the *Lancet* articles. We also make suggestions for how the chiropractic profession should most effectively help people with low back pain by implementing practices supported by high quality evidence.

## Background

Low back pain is a major global problem and it is getting worse [1]. An important series of articles in the journal *Lancet*, authored by world leading authorities on low back pain evidence, has drawn international attention to how enormous the problem is. The *Lancet* authors also discussed how low back pain is being poorly managed by healthcare systems around the globe, including emerging issues in low and middle-income countries [2–4]. This commentary will summarise the main findings of these *Lancet* papers, and provide some suggestions for how the chiropractic profession should respond to the global challenge that is low back pain.

## Lancet papers

In the first review paper, Jan Hartvigsen and colleagues [3] discussed the complexity of low back pain and the factors that contribute to it. They summarised the evidence base for the multiple factors known to cause or aggravate back pain, such as psychological, social, and biophysical factors, comorbidities, and pain-processing mechanisms. They argued that for the vast majority of people with low back pain, it is currently not possible to accurately identify the specific nociceptive source of pain. Hartvigsen and colleagues made a call for future research to “identify cost-effective and context-specific strategies” to better manage people with low back pain.

In the second review paper, Nadine Foster and colleagues [4] outlined the poor quality evidence base underpinning management of low back pain, and highlighted the lack of research into prevention of low back pain. They summarised the treatment recommendations from recent evidence-based clinical practice guidelines, including the application of a biopsychosocial framework, first line non-pharmacological care, and psychological approaches for people with persistent pain and disability. They also stated that guidelines recommend prudent use of medication, imaging, and surgery. They also highlighted the large gap between what is known, and what is actually occurring, in healthcare practices for people who seek care; many of these people receive inappropriate imaging, and treatments that are not helpful, or even harmful, such as rest, opioids, spinal injections, and surgery. Foster and colleagues provided recommendations for potential solutions to the current healthcare problems acknowledging that the evidence underpinning these solutions is inadequate and that more research is required to justify their widespread implementation.

The final paper was a commentary and “call for action” by Rachelle Buchbinder and colleagues [2]. These authors argued that low back pain needs to be prioritised, together with other musculoskeletal conditions, as a public health problem, particularly in low and middle-income countries. They suggested a way forward but also highlighted issues that may impede progress, including political challenges, such as increasing the recognition of the effects and burden of back pain by policy makers, and healthcare challenges, such as changing culture and changing clinician behaviour. They urged organisations such as the World Health Organisation to take action in an attempt to reduce increasing and costly effects of disabling low back pain.

## What do these papers mean for the chiropractic profession?

Providing care to people with low back pain is core business for the chiropractic profession, and low back pain is the most common presenting symptom to chiropractors in all parts of the globe [5]. The main message of these papers support an evidence-based chiropractic approach as a reasonable first line approach for patients with low back pain. In recent years there has been a major shift in thinking for the recommended management of low back pain, moving from a traditional biomedical model, towards a patient-centred biopsychosocial approach. Recent evidence-based guidelines have advocated the latter, recommending non-pharmacological approaches as first line treatment; pharmacological treatments are only recommended if non-pharmacological approaches are not providing adequate improvement [6–8]. Chiropractors are well placed to provide evidence-based non-pharmacological care for their patients with low back pain, including advice about physical activity, applying judicious manual therapy, education that supports self-management, and a graded return to normal activities and exercise.

However, there are also messages in the *Lancet* series that are challenging for some existing chiropractic practices, including the continued overuse of imaging, and treatment strategies that promote ongoing passive care. Although chiropractors are well placed to provide non-pharmacological treatment, some chiropractors continue to provide care that is contrary to guideline recommendations by ordering too many x-rays [9–12], over-servicing patients by providing services that promote ongoing passive care [13, 14], and providing treatments that are not supported by evidence [9].

Chiropractors are well placed to undertake the research that will result in better outcomes for people with low back pain. However, the profession continues to be under-represented in terms of numbers of chiropractors actively involved in research, and has a small research output compared to many other healthcare professions [15–17]. Some initiatives are in place to remedy this situation (for example [18]), but there is still much work to be done. Further, profession-specific funding bodies exist to build research infrastructure by stimulating healthcare research relevant to chiropractors. However, in our experience, some research funding bodies in the chiropractic profession seem more interested in promoting chiropractic research into the role of “subluxation” as it relates to health, or similar concepts. We have each submitted research funding applications to these funding bodies, only to have feedback that the low back pain problem is “already solved”. On the contrary, greater investment by the chiropractic profession in high quality research to address the societal burden of low back pain is urgently needed.

Chiropractors have much to offer as the healthcare system transforms to accommodate more patient-centred evidence-based biopsychosocial approaches. The chiropractic profession needs to be more integrated into mainstream healthcare to be a major player at the table as these initiatives recommended in the *Lancet* series are implemented.

## Conclusions

Our low back pain “call to action” for the chiropractic profession is to get our house in order. In our opinion, nothing is more relevant to chiropractors than people with low back pain, and the evidence clearly shows that we can do a better job for the millions of people who experience this potentially debilitating condition every year. Chiropractors in clinical practice need to provide higher quality care in line with recommendations from evidence-based clinical practice guidelines.

The chiropractic profession is perfectly placed to be a major player in providing a part of the solution to the global challenge of low back pain [19]. But the profession has been shut out of this role in most countries around the world due to, amongst many other things, internal political conflict, a lack of political will, and a minority of chiropractors who provide non-evidence-based approaches [20]. The profession needs to invest heavily to support chiropractors who wish to undertake high quality research directed at solving this major global problem.

## References

[1] Vos T, Flaxman AD, Naghavi M, Lozano R, Michaud C, Ezzati M (2012). Years lived with disability (YLDs) for 1160 sequelae of 289 diseases and injuries 1990-2010: a systematic analysis for the global burden of disease study 2010. Lancet.

[2] Buchbinder R, van Tulder M, Oberg B, Costa LM, Woolf A, Schoene M (2018). Low back pain: a call for action. Lancet.

[3] Hartvigsen J, Hancock MJ, Kongsted A, Louw Q, Ferreira ML, Genevay S (2018). What low back pain is and why we need to pay attention. Lancet.

[4] Foster NE, Anema JR, Cherkin D, Chou R, Cohen SP, Gross DP (2018). Prevention and treatment of low back pain: evidence, challenges, and promising directions. Lancet.

[5] Beliveau PJH, Wong JJ, Sutton DA, Simon NB, Bussieres AE, Mior SA (2017). The chiropractic profession: a scoping review of utilization rates, reasons for seeking care, patient profiles, and care provided. Chiropr Man Therap.

[6] NICE (2016). Low back pain and sciatica in over 16s: assessment and management. National Institute for Health and Care Excellence (NICE) Clinical Guidelines, 2016.

[7] Stochkendahl MJ, Kjaer P, Hartvigsen J, Kongsted A, Aaboe J, Andersen M (2018). National Clinical Guidelines for non-surgical treatment of patients with recent onset low back pain or lumbar radiculopathy. Eur Spine J.

[8] Qaseem A, Wilt TJ, McLean RM, Forciea MA (2017). Clinical guidelines Committee of the American College of P. Noninvasive treatments for acute, subacute, and chronic low back pain: a clinical practice guideline from the American College of Physicians. Ann Intern Med.

[9] Walker BF, French SD, Page MJ, O'Connor DA, McKenzie JE, Beringer K (2011). Management of people with acute low-back pain: a survey of Australian chiropractors. Chiropr Man Therap.

[10] Bussieres AE, Sales AE, Ramsay T, Hilles S, Grimshaw JM (2013). Practice patterns in spine radiograph utilization among doctors of chiropractic enrolled in a provider network offering complementary care in the United States. J Manip Physiol Ther.

[11] Ammendolia C, Cote P, Hogg-Johnson S, Bombardier C (2007). Do chiropractors adhere to guidelines for back radiographs? A study of chiropractic teaching clinics in Canada. Spine.

[12] Jenkins HJ (2016). Awareness of radiographic guidelines for low back pain: a survey of Australian chiropractors. Chiropr Man Therap.

[13] Morgan DJ, Dhruva SS, Wright SM, Korenstein D (2016). 2016 update on medical overuse: a systematic review. JAMA Intern Med.

[14] Heath I (2014). Role of fear in overdiagnosis and overtreatment--an essay by Iona heath. BMJ.

[15] French SD, Beliveau PJH, Bruno P, Passmore SR, Hayden JA, Srbely J, et al. Research priorities of the Canadian chiropractic profession: a consensus study using a modified Delphi technique. Chiropr Man Therap. 2017;25:38. 10.1186/s12998-017-0169-4 PMC5727882 29255593

[16] Rubinstein SM, Bolton J, Webb AL, Hartvigsen J (2014). The first research agenda for the chiropractic profession in Europe. Chiropr Man Therap.

[17] Stuber K, Kawchuk G, Bussieres A (2017). Research resource environment in Canada. Gathering knowledge in advance to inform chiropractic research priorities. J Can Chiropr Assoc.

[18] Adams J, Kawchuk G, Breen A, De Carvalho D, Eklund A, Fernandez M (2018). Leadership and capacity building in international chiropractic research: introducing the chiropractic academy for research leadership (CARL). Chiropr Man Therap.

[19] Schneider M, Murphy D, Hartvigsen J (2016). Spine care as a framework for the chiropractic identity. J Chiropr Humanit.

[20] Bussieres AE, Al Zoubi F, Stuber K, French SD, Boruff J, Corrigan J (2016). Evidence-based practice, research utilization, and knowledge translation in chiropractic: a scoping review. BMC Complement Altern Med.

